# Antibody response in children with multisystem inflammatory syndrome related to COVID-19 (MIS-C) compared to children with uncomplicated COVID-19

**DOI:** 10.3389/fimmu.2023.1107156

**Published:** 2023-03-15

**Authors:** Anaïs Thiriard, Benjamin Meyer, Christiane S. Eberhardt, Natasha Loevy, Serge Grazioli, Wafae Adouan, Paola Fontannaz, Fabienne Marechal, Arnaud G. L’Huillier, Claire-Anne Siegrist, Daphnée Georges, Antonella Putignano, Arnaud Marchant, Arnaud M. Didierlaurent, Geraldine Blanchard-Rohner

**Affiliations:** ^1^ Institute for Medical Immunology, Université Libre de Bruxelles, Brussels, Belgium; ^2^ Centre for Vaccinology, Department of Pathology and Immunology, University of Geneva, Geneva, Switzerland; ^3^ Pediatric Platform for Clinical Research, Department of Woman, Child and Adolescent Medicine, Geneva University Hospitals and Faculty of Medicine, University of Geneva, Geneva, Switzerland; ^4^ Division of Neonatal and Pediatric Intensive Care, Geneva University Hospitals and Faculty of Medicine, Geneva, Switzerland; ^5^ Pediatric Infectious Diseases Unit, Geneva University Hospitals and Faculty of Medicine, Geneva, Switzerland; ^6^ Laboratory of Enzymology and Protein Folding, Centre for Protein Engineering, InBioS, University of Liège, Liège, Belgium; ^7^ Pediatric Immunology and Vaccinology Unit, Children’s Hospital of Geneva, Geneva University Hospitals and Faculty of Medicine, Geneva, Switzerland

**Keywords:** MIS-C multisystem inflammatory syndrome in children, SARS-CoV-2, antibody response, functional antibody, common coronavirus, commensals

## Abstract

**Objectives:**

To comprehensively analyze the quality of the antibody response between children with Multisystem inflammatory syndrome (MIS-C) and age-matched controls at one month after SARS-CoV-2 exposure, and infected in the same time-period.

**Methods:**

Serum from 20 MIS-C children at admission, and 14 control children were analyzed. Antigen specific antibody isotypes and subclasses directed against various antigens of SARS-CoV-2 as well as against human common coronavirus (HCoVs) and commensal or pathogenic microorganisms were assessed by a bead-based multiplexed serological assay and by ELISA. The functionality of these antibodies was also assessed using a plaque reduction neutralization test, a RBD-specific avidity assay, a complement deposition assay and an antibody-dependent neutrophil phagocytosis (ADNP) assay.

**Results:**

Children with MIS-C developed a stronger IgA antibody response in comparison to children with uncomplicated COVID-19, while IgG and IgM responses are largely similar in both groups. We found a typical class-switched antibody profile with high level of IgG and IgA titers and a measurable low IgM due to relatively recent SARS-CoV-2 infection (one month). SARS-CoV-2-specific IgG antibodies of MIS-C children had higher functional properties (higher neutralization activity, avidity and complement binding) as compared to children with uncomplicated COVID-19. There was no difference in the response to common endemic coronaviruses between both groups. However, MIS-C children had a moderate increase against mucosal commensal and pathogenic strains, reflecting a potential association between a disruption of the mucosal barrier with the disease.

**Conclusion:**

Even if it is still unclear why some children develop a MIS-C, we show here that MIS-C children produce higher titers of IgA antibodies, and IgG antibodies with higher functionality, which could reflect the local gastro-intestinal mucosal inflammation potentially induced by a sustained SARS-CoV-2 gut infection leading to continuous release of SARS-CoV-2 antigens.

## Introduction

All children can be infected by SARS-CoV-2, independently of their age (1, 2). In contrast to adults, children often manifest non-specific symptoms similar to other pediatric infections, such as fever, cough and gastro-intestinal symptoms (3, 4). The majority of children present with asymptomatic or pauci-symptomatic infections (2).

In the spring 2020, during the peak of the first wave of the coronavirus disease 2019 (COVID-19) pandemic, clusters of children with severe hyperinflammatory syndromes suggestive of atypical Kawasaki disease (KD) or toxic shock syndrome were reported in Europe and the U.S. These cases occurred at around 2-4 weeks following SARS-CoV-2 infection or exposure (98% and 2% respectively, CDC last report 31^st^ October 2022), confirmed by a positive COVID-19 serology or reverse-transcription (RT-) polymerase chain reaction (PCR) (5–8). This syndrome was named Multisystem Inflammatory Syndrome in Children (MIS-C) by The World Health Organization (WHO), or Pediatric Inflammatory Multisystem Syndrome temporally associated with SARS-CoV-2 (PIMS-TS) by the UK Royal College of Pediatrics and Child Health. MIS-C affects < 1% of children infected with SARS-CoV-2, with an estimated incidence between 3/10 000 infected and 2/100 000- infected children (9–11). MIS-C remains a rare complication of SARS-CoV-2 infection, however, it can be very severe and associated with a high risk of hospitalization in the intensive care as well as with decreased cardiac heart function and requires a rapid recognition of the syndrome to set up treatment. Therefore, it is important to understand the causes of this syndrome in order to apply public health preventive strategies. MIS-C affects healthy children, predominantly male, with a median age of 8-11 years (range 1-20 years). At the beginning of the pandemic, Hispanics/Latinos and Blacks were overrepresented among MIS-C cases, while later on all ethnicities were affected, except from Asia, in contrast to KD (5–8). Children typically present with persistent high fever, gastrointestinal symptoms and with a continuum of severity ranging from mild symptoms to multi-organ failure with lethargy, hypotension, and shock, which require fluid resuscitation, inotropic support and sometimes also mechanical ventilation and extracorporeal membrane oxygenation (7, 11–13). Children with MIS-C have elevated inflammatory markers including C-reactive protein (CRP), procalcitonin, erythrocyte sedimentation rate (ESR), ferritin, and also cytokines such interferon γ and tumor necrosis factor. They have an increased white blood cell count with concomitant profound lymphopenia, and neutrophilia. Children can develop coronary dilatation in 8-26% of cases (12, 14–16).

The physiopathology of the MIS-C has been widely studied, but there is still no clear explanation on the exact cause leading to MIS-C development in some children. It has been reported that the various symptoms of MIS-C reflect local vasculitis and inflammation of the affected organs. Similarly to KD, MIS-C is triggered by infection but the inflammatory response is more intense and the cytokines profile different in children with MIS-C compared to children with KD; in KD, there is a robust increase in IL-17A, while MIS-C, is characterized by a robust increase in IL-1, a cytokine produced by endothelial cells that are damaged by autoantibodies and complement (17). Auto-antibodies have been observed in MIS-C, such as auto-antibodies directed against the endothelial, gastrointestinal and immune cells (17, 18). These auto-antibodies may form immune complexes and/or trigger a cell mediated attack against host tissues (18). Auto-antibodies are thought to be induced by cross-reactivity between the virus and host structures. In addition, children with MIS-C have mesenteric adenitis and ileitis which could reflect a pattern of gastro-intestinal infection by SARS-CoV-2, given that enterocytes have been shown to be infected by the virus (6, 8, 19). Altogether, the hyperinflammatory process and local vasculitis could be due to a direct effect of the virus on infected organs or an indirect environmental trigger in genetically predisposed individuals. As MIS-C occurs two to four weeks after acute SARS-CoV-2 infection, a role for adaptive immune effectors is pointed out. Limited data suggest that the humoral immune response in MIS-C patients is different compared to children with uncomplicated COVID-19. One study reported that MIS-C patients develop antibodies that effectively neutralize SARS-CoV-2 *in vitro*, but they have lower levels of IgM and higher levels of IgA and anti-spike IgG (20). Another study has reported that high titers of SARS-CoV-2 spike receptor-binding domain (RBD) IgG antibodies in children with MIS-C that correlated with full-length spike (S) IgG antibodies, nucleocapsid (N) protein antibodies and neutralizing antibodies (21). All MIS-C children also had RBD IgM antibodies, suggesting recent SARS-CoV-2 infection. RBD IgG titers correlated also with parameters of disease severity such as the erythrocyte sedimentation rate and the hospital and ICU lengths of stay (21). Comparison of adults with severe disease and children with and without MIS-C showed that children produced mainly IgG antibodies specific for the S protein but not for the N protein, while the adults generated IgG, IgM and IgA antibodies against the S and N proteins. In addition, the children had a reduced neutralizing activity compared to the adults (22).

In this study, we wanted thus to analyze the amplitude and the effectiveness of the humoral immune responses of children with MIS-C and children with uncomplicated COVID-19 at approximately one month post infection. This study offers a unique deep analysis of the antibody response, at the same time point between the MIS-C children and the children with uncomplicated COVID-19. Indeed, the control children with uncomplicated COVID-19 are taken from a study, which was designed to follow-up contacts of recently diagnosed individuals to increase the chance of studying infection in the first days (23). Therefore, the control children had blood sampling from the onset of infection to its resolution, with follow-up samples available at one-month post-infection, which corresponds to the time interval at which MIS-C usually occurs. In addition, both the control children and the MIS-C children had been infected during the same time period, which corresponded to when the original strain and the antigenically similar alpha variant of SARS-CoV-2 circulated in Switzerland.

## Materials and methods

### Study population

This prospective observational study included all pediatric patients (aged < 18 years) hospitalized at the Geneva University Hospitals with a confirmed diagnosis of MIS-C according to World Health Organization (WHO) case definition between April 2020 and May 2021. The study was approved by the local Ethics committee of Geneva (CCER 2020 00835), and written informed consent was obtained from all participants and their legal representatives. Demographic, laboratory, and physiological characteristics were documented for each participant at hospital admission and during hospital stay (see **Table 1**). One additional blood sample was collected at admission during other sampling as part of standard of care. In addition, the control children with uncomplicated COVID-19 were recruited from the study “Understanding Covid”, a single-center prospective observational study conducted at the Geneva University Hospital, to examine early virological and early and late immunological responses among SARS-CoV-2-infected adults and children (23). The study was approved by the Geneva Cantonal Ethics Commission (CCER 2020-00516), and written informed consent was obtained from all participants and their legal representatives. The control children had longitudinal blood sampling at various times after infection, up to one year.

**Table 1:** Clinical characteristics among MIS-C children.

	MISC (N=20)
SARS-CoV-2	N
PCR (pos/neg)	3/17
Serology (pos/neg)	17/0
Cells count	Median (IQR)
WBC (g/l)	7.9 (5.5-11.7)
Neutrophiles (g/l)	4.9 (3-7.2)
Lymphocytes (g/l)	0.5 (0.3-1)
Inflammatory markers	Median (IQR)
CRP (mg/l)	209.7 (171.9-268)
Procalcitonine (µg/l)	5.94 (1.7-13.1)
ESR (mm/hr)	52 (29-64)
Ferritine (µg/l)	654.5 (346.5-1205.5)
Cytokines	Median (IQR)
TNFa (pg/ml)	20 (10.9-24.3)
IL6 (pg/ml)	91.6 (22.6-206.8)
IL8 (pg/ml)	45 (16.6-63.9)
IL1Ra (pg/ml)	10000 (6807.5-22792.5)
MCP1 (pg/ml)	624.95 (251.4-1226.8)
IL1b (pg/ml)	1.1 (1-2.9)
IL10 (pg/ml)	54.6 (8-278.8)
IL17 (pg/ml)	9.5 (3.8-26.1)
Treatment	N
IVIG (yes/no)	16/4
Corticoïdes (yes/no)	16/4
Anti-IL1 (yes/no)	5/15
Anti-IL6 (yes/no)	3/17
Inotropic support (yes/no)	12/8
Mechanical ventilation (yes/no)	2/18
Non invasive ventilation (yes/no)	3/17
Symptoms	N or Median (IQR)
Acute kidney injury (yes/no)	7/13
Coronary artery issue (yes/no)	4/16
Days of fever	5 (3.8-6)
Days of hospitalization	7 (6-10)

### Antigens

The following wild-type SARS-CoV-2 antigens were used: trimerized spike protein (triS) (EPFL, protein production facility), receptor-binding-domain of the spike protein (RBD) (SinoBiological #40592-VNAH), sub-unit S1 (SinoBiological #40591-V08H), and the sub-unit S2 (SinoBiological #40590-V08B) and nucleocapside protein (N) (Prospec-Tany #sars-040 or kindly provided by Andre Matagne from University of Liege, Belgium). The following HCoV spike proteins were used: trimerized spike protein, S1 and S2 from the betacoronaviruses HKU1 and OC43 (EPFL, protein production facility, SinoBiological #40021-V08H, #40021-V08B, #40607-V08H1, #40607-V08B1), and spike from the alphacoronaviruses NL63 (SinoBiological #40604-V08B). The following commensal and pathogenic antigens were used: total extract, TotExt, and a mixture of surfactome and secretome, SurSec, (kindly provided by Anne Botteaux from BacMol Lab, Belgium) from the *E. coli* K12 MG1655 and *S. aureus* (ATCC #29213), flagellin from *E. coli*, FliEc (Cusabio #CSB-EP300968ENVe1), flagellin from *B. subtilis*, FlaBs (*In vivo*gen # Tlrl-PBSFla), flagellin from *P. aeruginosa*, FlaPa (*In vivo*gen, #tlrl-pafla), zymozan from *S. cerevisiae*, ZymSc (*In vivo*gen, #tlrl-zym), heat killed and sonicated *C. albicans*, hkCa (*In vivo*gen, #tlrl-hkca), beta hemolysin from *S. aureus* (βH) (in collaboration with CER group). The tetanus toxoid from *C. tetani* (MassBiologics) was included in the analysis as a control antigen.

### Titers of SARS-CoV-2, HCoVs and commensal and pathogen specific antibodies

Titers of antigen-specific antibody isotypes and subclasses were assessed using a 96-well based customized multiplexed Luminex assay. Briefly, antigens were coupled by covalent NHS-ester linkages *via* EDC and NHS (Pierce #77149 and #24520, respectively) to fluorescent carboxyl modified microspheres (Luminex). Antigen-coupled microspheres were incubated 2 h at room temperature (RT) at 700 rpm orbital shaking with serum samples at appropriate dilution: 1:1000 for IgG and IgG1, 1:500 for IgG3, 1:200 for IgM, IgG2, IgA, IgA1 and IgA2, 1:10 for IgG4 titration. Antigen-specific antibody titers were detected using 0.65 µg/ml of PE-coupled detection antibody for each isotype and subclass, including IgM (Southern Biotech #9020-09), IgG (Biolegend #409304), IgG1 (Southern Biotech #9052-09), IgG2 (Southern Biotech #9070-09), IgG3 (Southern Biotech #9210-09), IgG4 (Southern Biotech #9200-09), IgA (Southern Biotech #2050-09), IgA1 (Southern Biotech #9130-09) and IgA2 (Southern Biotech #9140-09). Antigen-antibody reactions were read on a BioPlex-200 (Bio-Rad) after 1 h incubation and the results were expressed as median fluorescence intensity (MFI).

### Enzyme-linked immunosorbent assay (ELISA)

Anti-S1 and anti-N antibody titers were measured using a standard ELISA protocol. Briefly, ELISA (Nunc) plates were coated with SARS-CoV-2 S1 (SinoBiological #40591-V08H), N (Prospec-Tany #sars-040) 1 μg/mL or without antigen (PBS) over night at 4°C. Plates were washed with PBS + 0.05% Tween20 (PBS-T) and blocked with PBS-T + 20%BSA for 1 h. Afterwards plates were incubated for 1 h at 37°C with 2-fold serial diluted sera, washed, and incubated with anti-human IgG peroxidase-conjugated antibody (Jackson, #109-036-098) for 1 h at 37°C. Plates were revealed using TMB substrate and HCl and read with an ELISA reader (450nm, SoftMax Pro Version 6.2.2). On each plate, a 2-fold serial dilution of a reference serum (arbitrary units: 100) was applied and used for standardization. Optical density (OD) units from PBS coated wells were subtracted and corrected ODs from sample dilutions were interpolated on the standard curve using a 4 parameter logistic (PL) regression model with SoftMax Pro Version 6.2.2. Dilutions within the linear range of the assay were used to calculate final AU/ml for each serum sample.

Antigen RBD concentration in serum from MIS-C and control children was measured by a standard ELISA kit (abcam # ab284402). According to the manufacturer’s instructions, serum was diluted 2-fold and 4-fold and OD were measured at 450nm. OD units from PBS coated wells were subtracted and corrected ODs from sample dilutions were interpolated on the standard curve using a 4-parameter logistics (PL) regression model with GraphPad Prism (v9.5.1). The mean OD was calculated for all samples.

### Plaque reduction neutralization test (PRNT)

Vero E6 cells were seeded at a density of 4x10^5 cells/ml in 24-well cell culture plates 1 day before and incubated over night at 37°C, 5% CO2. All sera were inactivated at 56°C for 30min and 2-fold dilution series in Opti-Pro serum free medium (Gibco, # 12309019) (volume: 220µl for each dilution) were prepared starting with a 1:5 (final dilution 1:10 after addition of virus) dilution. Recombinant SARS-CoV-2 strain Wuhan-Hu-1 (kindly provided by V. Thiel, University of Bern) was diluted to a concentration of 500 PFU/ml in Opti-Pro and 220µl of virus was added to the serum dilutions. Virus serum mixtures were incubated at 37°C for 1 h. Virus without serum was used as an infection control and medium without virus was used as a negative control. All samples were run in duplicate and for each neutralization experiment, a reference serum was used to insure reproducibility between different experiments. Medium was removed and Vero E6 cells were washed 1x with PBS before inoculation with virus serum mixture (200µl/well) for 1 h at 37°C, 5% CO2. Afterwards, the inoculum was removed and 500µl of a high viscosity overlay medium (DMEM + 1% Glutamax + 1% Penicillin Streptamycin + 10% FBS + 1.2% Avicel) was applied to each well. After incubation for 40 h at 37°C, 5% CO2, the overlay medium was removed, cells were fixed in 6% formaldehyde solution for 1 h, plates were washed 1x with PBS and finally stained with crystal violet for 20 min. Plaques were counted in wells inoculated with virus-serum mixtures and compared to plaque counts in infection control wells (virus without serum). The 90% reduction endpoint titers (PRNT90) were calculated by fitting a 4-PL curve with variable slope to the plaque counts of each serum/plasma using GraphPad Prism version 9.4.1.

### SARS-CoV-2 RBD-specific antibody avidity

Bio-layer interferometry measurements were performed with an Octet HTX instrument (FortéBio) using AR2G biosensors. Data analyses were performed using FortéBio Data Analysis 9.0 software. Kinetic assays were performed at 25-30˚C at a sample plate agitation speed of 1000 rpm. Sensors were first activated by immersion in a solution containing 20 mM EDC and 10 mM s-NHS. Then, 0.05 mg/ml of RBD antigen in 10 mM sodium acetate pH6 was loaded for 600sec. After antigen loading, the biosensors were immersed in a solution of 1 M ethanolamine pH8.5 to prevent non-specific interactions. Antigen loaded AR2G sensors were first dipped in PBS to establish a baseline time curve, and then immersed for 10 min in wells containing purified serum IgG at three different dilutions (3-5-8x). Following IgG association, dissociation was monitored for 600 sec in PBS. Negative controls included ligand without IgG and IgG without ligand. Kinetic parameters were determined by global fitting of the association and dissociation phases of the binding curves according to a 1:1 binding model.

### Complement deposition assay

Antibody-Dependent Complement Deposition (ADCD) was quantified using 96-well based customized multiplexed Luminex assay. Bulk IgG was purified and separated from other serum proteins using Melon Gel resin according to the manufacturer’s instructions (Thermo Scientific #45208). Purified IgG at 1:75 dilution for IgG anti-RBD and at 1:150 dilution for IgG anti-S1, -S2, -S, -NP and -TT, was incubated with antigen-coupled beads for 2 h at 37°C on 700 rpm orbital shaker. After incubation, each sample was incubated with human complement serum (Sigma #S1764) at a concentration of 1:50 at 37°C for 30 min. Samples were then incubated at RT for 30 min with biotinylated monoclonal anti-human C3d (Quidel #207) at 1 µg/ml final concentration. Finally, 1 µg/ml of Streptavidin-RPE (Prozyme #PJ31S) was added to each well and incubated at 37°C in the dark for 1 h. Complement deposition was determined on a BioPlex-200 equipment (Bio-Rad) and measured as MFI. Assays performed without IgG and with heat-inactivated human complement serum were used as negative controls.

### Antibody-dependent neutrophils phagocytosis (ADNP)

Whole blood from healthy donors was treated with ACK lysis buffer (ammonium chloride 155mM, potassium bicarbonate 10mM, N2EDTA 0,1mM, pH7,2) to lyse the red blood cells. Briefly, 9 parts of ACK lysis buffer was added to 1 part of the blood, mixed gently by inverting the tube several times and incubated on ice for 10 min. Followed by centrifugation at 450 g, 5 min and 4°C. The supernatant was discarded and pelleted cells were washed twice with chilled complete media (RPMI 1640, 10% FBS, L-Glut 2mM, penicillin/streptomycin 100/100, NEAA) by centrifuging at 450 g for 5 min at 4°C. Cells were resuspended in complete medium at 50 0000 cells/ml and left on ice until usage. Prior the assay, recombinant antigens were biotinylated using EZ-Link NHS-LC-LC-Biotin (Thermo #21343) for 30 min at 37°C with the following molar ratio: 1 mole of antigen for 50 moles of biotin. The biotinylated-antigens were then purified using zeba spin desalting columns (Thermo #89883). Then, each biotinylated antigen was coupled, independently, to FluoSpheres NeutrAvidin beads (Thermo #F8776) for 2 h at 37°C. Based on optimization experiments, the volume ratio of biotinylated-antigen used to be incubated with the beads was determined as 1 volume of bead for 3 volumes of biotinylated antigen. Antigen-conjugated beads were then blocked (PBS-1X, 0.1% BSA) and diluted 100-fold in this buffer. Antigen-coated beads were incubated with purified IgG for 2 h at 37°C. The acquisition was done by a LSR-Fortessa (BD) with the following voltages: FSC=150V, SSC=230V, FITC=270. Results were expressed as phagocytic score: FITC+frequency*FITC+mean*10-4. The purity of neutrophils was confirmed by staining with CD66b (BioLegend #305102).

### Statistics

Comparisons of antibody titers, ADCD and ADNP between MIS-C and uncomplicated COVID-19 groups were performed on log10-transformed data using unpaired two-tailed t-test followed by correction for multiple comparisons by controlling the false discovery rate at 5% using the Benjamini-Hochberg method. Normalized values for neutralization activity and ELISA titers were log10-transformed and normalized avidity measures and ELISA RBD-concentration were analyzed with unpaired two-tailed t-test. Correlation matrices depicted p values calculated by a two-tailed Spearman test. Dot plots presented a regression line, a 95% confidence interval dashed line and r and p values from a two-tailed Spearman test were calculated. Statistical differences were depicted as following: not significant (ns) p>0.05, * 0.01<p ≤ 0.05, ** 0.001<p ≤ 0.01, *** 0.0001<p ≤ 0.001, **** 0.0001≤p. Statistical analyses were performed using R or GraphPad Prism software, version 9.4.1.

## Results

### Study cohort

In our study we investigated the humoral immune response in children with MIS-C and a control group of children that had a RT-PCR confirmed SARS-CoV-2 infection but did not develop MIS-C. We included 20 children that were hospitalized with a diagnosis of MIS-C between 14.04.2020 and 27.04.2021 with a history of mild or asymptomatic SARS-CoV-2 infection. As MIS-C typically develops 3-4 weeks after infection only three children in our study were still PCR positive but all children had antibodies against SARS-CoV-2. As a control group we included 14 children without MIS-C that had COVID-19 during a similar time period (13.05.2020-15.02.2021) and were recruited as part of another observational study (23). Controls were slightly younger than children with MIS-C (median age 9 years (IQR=6.5) for control children and 12 years (IQR=5) for the MIS-C group). There were slightly more males in the MIS-C group (85%) compared to the control group (64.3%) but the difference was not significant either (p=0.2278) (**Table 2**). Based on their timing of infection, both groups were either infected with ancestral SARS-CoV-2 strains circulating in 2020 or the antigenically closely related Alpha variant. The ethnicity of children in both groups was predominantly Caucasian, followed by African, mixed Caucasian-African and other ethnicities (**Table 2**).

**Table 2:** Patient characteristics.

	Controls	MIS-C	Statistics
Number	14	20	
Sampling dates T_0	13.05.2020-15.02.2021	14.04.2020-27.04.2021	
Age			
Median (IQR)	9 (6-10) IQR=6,5	12 (9-14) IQR=5	p=0.0715
Sex			
Female	5 (35.7%)	3 (15.0%)	p=0.2278
Male	9 (64.3%)	17 (85.0%)	
Ethnicity			
Caucasian	7 (50.0%)	13 (65.0%)	
African	2 (14.3%)	5 (25.0%)	
Arab	0 (0%)	1 (5.0%)	
Hispanic	1 (7.1%)	1 (5.0%)	
Mixed Caucasian-African	3 (21.4%)	0 (0%)	
Mixed Caucasian-Asian	1 (7.1%)	0 (0%)	

### MIS-children have higher IgA antibody levels compared to children with uncomplicated COVID-19

**Figure 1:**
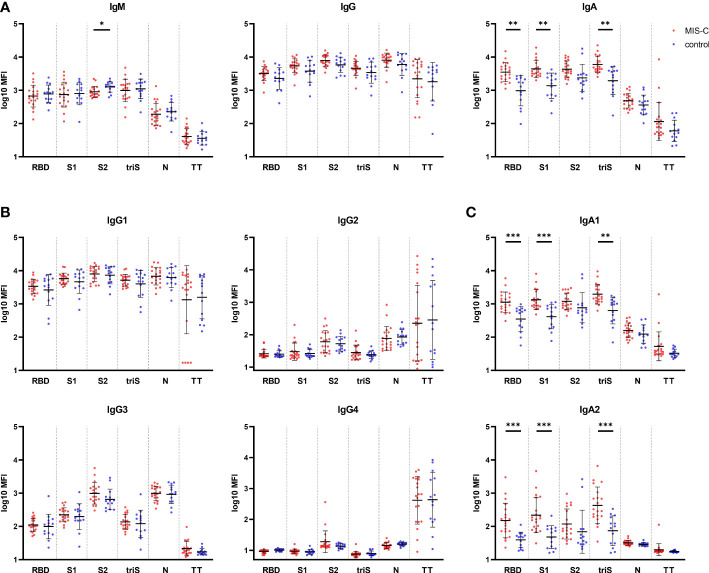
Binding antibody response against SARS-CoV-2 in MIS-C and control children. **(A)** IgM (left panel), IgG (middle panel) and IgA (right panel) antibody levels against SARS-CoV-2 receptor binding domain (RBD), S1 and S2 domain of the spike protein, trimerized full length spike (triS) and nucleocapsid protein (N) as well as a tetanus toxoid (TT) in MIS-C (red dots) and control children (blue dots). Levels of IgG **(B)** and IgA **(C)** antibody subclasses against the same antigens. Antibody levels, measured using a bead-based multiplex platform, are expressed as median fluorescence intensity (MFI) and were log10 transformed. Results have been duplicated and mean and standard deviations are represented. * 0.01<p≤0.05, ** 0.001<p≤0.01, *** 0.0001<p≤0.001.

We first perform a comprehensive analysis of binding antibodies isotypes against various SARS-CoV-2 derived antigens, i.e. RBD, S1 and S2 domain of the trimerized spike (triS) and N, using a multiplex bead-based serological assay. The relative high level of IgM (**Figure 1A**, left panel) against spike (RBD, S1, S2 and triS) compared to TT confirmed that all children had recently been infected with SARS-CoV-2. We found no significant difference in IgM and IgG (**Figure 1A**, middle panel) between children with MIS-C and the control group for all antigens including TT, suggesting that the time between infection and blood sampling was similar in both groups. IgM directed against S2 (p=0.0458) was slightly higher in the control group but given the small difference observed, this finding is unlikely to be biologically relevant. These results were confirmed for IgG antibodies directed against S1 and N antigens using ELISA (**Figure S1**). In contrast to IgG and IgM, children who developed MIS-C had significantly higher IgA antibody responses (**Figure 1A**, right panel) directed against RBD, S1 and triS (p=0.0042), but not against S2 and N antigens compared to control children. To further characterize antibody responses, we also analyzed subclass IgG1,2,3 and 4, IgA1 and IgA2 against the same set of antigens. SARS-CoV-2 specific IgG response was mainly IgG1 as reported by others (23) and similar high levels of antibodies against each antigen were measured in both groups (**Figure 1B**). Low IgG2 titers against S2 and N antigens and a very weak IgG4 were measured. Interestingly, the relative titers of IgG3 were higher against S2 and N than against RBD, S1 or triS (**Figure 1B**), indicating a potential cross-reactivity between SARS-CoV-2 and other coronaviruses for the S2 domain and nucleocapsid antigens. Similar to total SARS-CoV-2 specific IgG and IgA responses, we detected higher IgA1 and IgA2 titers against RBD, S1 and triS (IgA1: p=0.0008, p=0.0006 and p=0.0012 respectively; IgA2: p=0.0005) in MIS-C compared to control children (**Figure 1C**). No significant difference was observed with S2 and N antigens. Collectively, our serological analysis shows that MIS-C children have a unique IgG and IgA profile.

### MIS-C children exhibit potent IgG functional response compared to children with uncomplicated COVID-19

**Figure 2:**
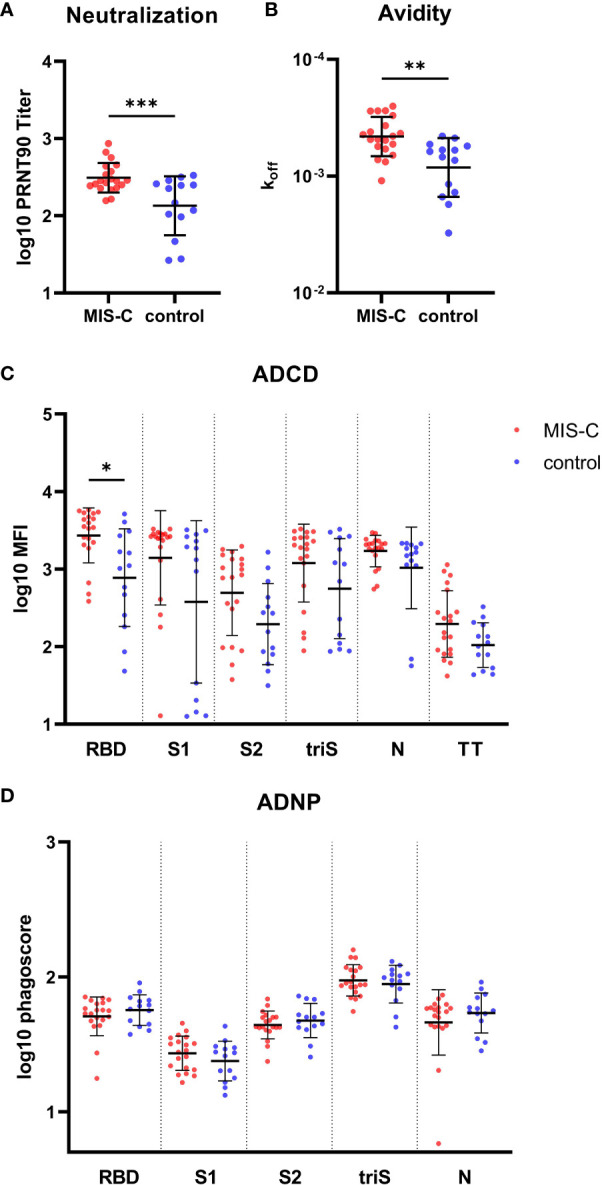
Functional IgG antibody response against SARS-CoV-2 in MIS-C and control children. **(A)** SARS-CoV-2 specific neutralizing antibodies determined by plaque reduction neutralization assay. **(B)** Avidity of IgG antibodies against SARS-CoV-2 receptor binding domain (RBD). Antibody dependent complement deposition (ADCD) **(C)** and antibody dependent neutrophil phagocytosis (ADCP) **(D)** directed against SARS-CoV-2 RBD, S1 and S2 domain of the spike protein, trimerized full length spike (triS) and nucleocapsid protein (N) as well as a tetanus toxoid (TT) in MIS-C (red dots) and control children (blue dots). Results of antibody neutralization titer, complement binding and phagoscores were log10 transformed. Results have been duplicated, means and standard deviations are represented for **(A, C, D)** and geometric means and geometric standard deviations for **(D)**. * 0.01<p≤0.05, ** 0.001<p≤0.01, *** 0.0001<p≤0.001.

Next, we investigated the difference in the functionality of the IgG antibody response between MIS-C and controls despite similarities in the amplitude of the response. Neutralizing antibodies were determined using gold standard plaque reduction neutralization assay. At the onset of MIS-C symptoms, we found a significantly higher neutralizing capacity of serum antibodies in MIS-C children compared to the control group (p=0.0009) (**Figure 2A**). Similarly, RBD-specific IgG antibodies had a significantly higher avidity in MIS-C children (p=0.005) (**Figure 2B**) that correlated with neutralization capacity (**Figure S2**). This effect was seen irrespective of the age of the children (data not shown). These results indicate that SARS-CoV-2 specific IgG antibodies have a higher functional capacity in MIS-C to bind and neutralize the virus compared to children with uncomplicated COVID-19.

To further delineate functional antibody responses, the capacity of IgG to activate the classical pathway of the complement cascade and neutrophil phagocytosis was measured for all antigens. There was a trend for higher complement activation in the MIS-C group (NP = 0.163, RBD = 0.0431, S = 0.1525, S1 = 0.1385 , S2 = 0.09675), with a significant difference measured for anti-RBD IgG (p=0.0431) (**Figure 2C**). No difference was observed for antibody-dependent neutrophils phagocytosis (ADNP) (**Figure 2D**). Taken together, MIS-C IgG antibodies displayed higher functional properties (i.e. higher neutralization activity, avidity and complement binding) as compared to children with uncomplicated COVID-19.

### The specific antibody profile in MIS-children is not due to difference in antibody response to common endemic coronaviruses

**Figure 3:**
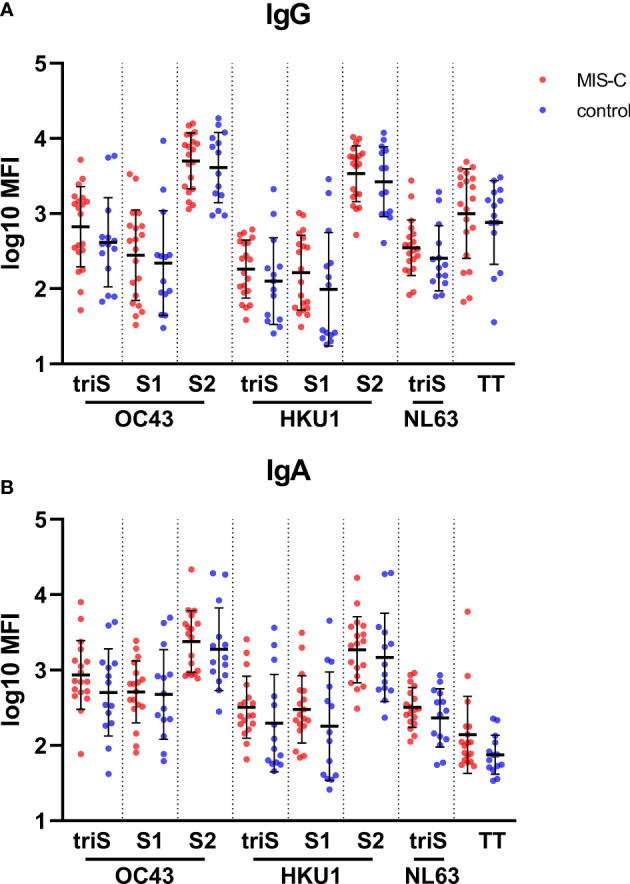
Binding antibody response against human common cold coronaviruses in MIS-C and control children. IgG **(A)** and IgA **(B)** antibody levels against trimerized full length spike (triS) as well as S1 and S2 domain of the spike protein of OC43, HKU1, and NL63 (trimerized spike only) human common cold coronaviruses as well as a tetanus toxoid (TT) in MIS-C (red dots) and control children (blue dots). Antibody levels, measured using a bead-based multiplex platform, are expressed as median fluorescence intensity (MFI) and were log10 transformed. Results have been duplicated and the mean and standard deviations are represented.

We hypothesized that a different response to common endemic coronavirus in MIS-C children could explain the difference in antibody quality observed in our cohort. We measured the IgG and IgA binding against antigens derived from human common cold coronaviruses (HCoVs) HCoV-OC43 and HCoV-HKU1, belonging to the genus beta-coronavirus as SARS-CoV-2, and from HCoV-NL63, a more distantly related alpha-coronavirus. No difference in IgG (**Figure 3A**) or IgA (**Figure 3B**) antibody levels against most of the antigens from the HCoV-OC43, HCoV-HKU1 and HCoV-NL63 was found between MIS-C and controls. Only a higher level of IgG and IgA against the S2 domain of the *beta-coronavirus* was measured, suggesting that the SARS-CoV-2 infection had boosted a pre-existing response to HCoVs. Moreover, the IgG and IgA responses towards these common cold viruses are not dependent on the age of the children (data not shown). These results indicate that a recent exposure or co-infection with HCoVs does not explain higher SARS-CoV-2 specific IgA titers in MIS-C.

### MIS-C children unveil an atypical IgA response to bacteria

Given the potential of SARS-CoV-2 to infect mucosal tissues and the potential association between MIS-C and gastrointestinal symptoms, we assessed IgA and IgG responses towards gut commensal *(E. coli K12 MG1655, B. subtilis)* and other pathogenic *(P. aeruginosa, S. cerevisiae, C. albicans, S. aureus*) microorganisms to see if a dysregulated response to the microflora could be implicated in the onset of MIS-C. Our hypothesis was that the inflammation associated to MIS-C might favorize translocation of microbiome components in the systemic circulation to trigger Ig production upon B cells stimulation. Therefore, we considered bacteria belonging to fila, families, genera and spp which are usually well represented among the commensal bowel microbiome in physiological conditions as well as in other pathologies, that, similarly to MIS-C, are characterized by mucosal and systemic inflammation (24, 25). Our purpose was also to investigate whether the nature of the producing source might affect the quality of humoral response.

Consequently, we wanted to compare Ig directed against poli-antigenic preparation extracted from gram-positive and gram-negative bacteria which contain a diverse protein composition including wall proteins and peptidoglycans. Our choice on *S. Aureus* and *E. Coli* was driven by their substantial representation in the bowel and respiratory microbiome. Indeed, these spp. belong to the Firmicutes Filum, which is well represented in the bowel microbiome in physiological conditions. Also, the proportion of Staphylococcaceae and Enterobacteriaceae families within the Firmicutes Filum is often increased in some pathological and inflammatory settings (26).

Next, we wanted to evaluate whether the poli-antigenic humoral response was comparable to the one directed against single antigens. Therefore, we directed our choice to highly immunogenic antigens, such as Hemolysins (27), or antigens that have been described as powerful triggers of the humoral response in other pathological inflammatory conditions, such as Flagellin, already reported as inducer of circulating IgG in inflammatory bowel disease (28).

Finally, we wanted to investigate if the humoral response against bacteria which are purely commensal was similar to the one against potentially pathogenic ones. Consequently, we decided to compare, after ruling out intra-assay competitive mechanisms, Ig directed against Flagellin isolated from B. Subtilis (commensal nonpathogenic spp.), E.Coli (potentially pathogenic spp.) and Ps. Aeruginosa (pathogenic one, usually very poorly represented in physiological conditions).

**Figure 4:**
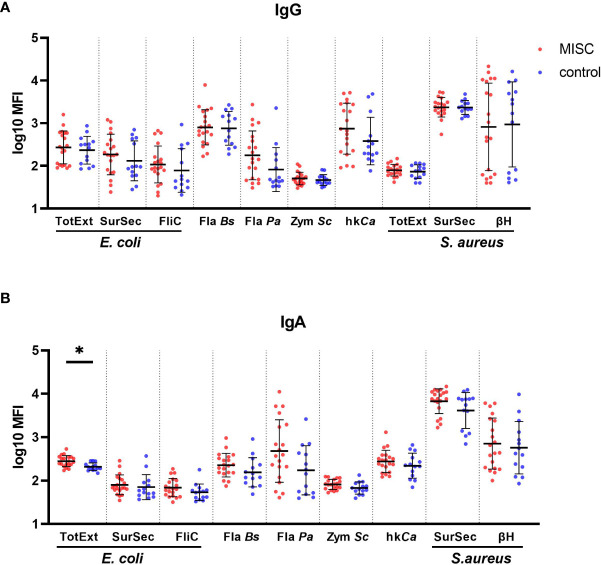
Binding antibody response against commensal and pathogenic microbes in MIS-C and control children. IgG **(A)** and IgA **(B)** antibody levels against total extract (TotExt), surfactome/secretome (SurSec) and flagellin (FliC) from *Escherichia coli*, flagellin from *Bacillus subtilis* (Fla Bs) and *Pseudomonas aeruginosa* (Fla Pa), zymosan from *Saccharomyces cerevisiae* (Zym Sc), heat killed and sonicated *Candida albicans* (hkCa), as well as total extract (TotExt), surfactome/secretome (SurSec) and beta hemolysin (βH) from *Staphylococcus aureus* in MIS-C (red dots) and control children (blue dots). Antibody levels, measured using a bead-based multiplex platform, are expressed as median fluorescence intensity (MFI) and were log10 transformed. Results have been duplicated and the mean and standard deviations are represented. * 0.01<p≤0.05.

No significant difference for IgG titers was observed between MIS-C and children with uncomplicated COVID-19 (**Figure 4A**). The MIS-C group produced significantly higher levels of IgA against total extract from *E. coli* (p=0.0251) compared to the control group (**Figure 4B**). There was also a general trend for higher levels of IgG anti-*C. albicans* (p=0.373) and anti-flagellin from *P. aeruginosa* (p=0.373) and higher IgA responses towards anti-surface/secreted extract from *S. aureus* but without statistical significance (p=0.373). Taken together, these results suggest that children with MIS-C presented a modest IgA increase against mucosal commensal and pathogenic strains, reflecting a potential association between a disruption of the mucosal barrier with the disease.

### Lower spike antigen level in MIS-C children negatively correlate with spike specific IgA responses

**Figure 5:**
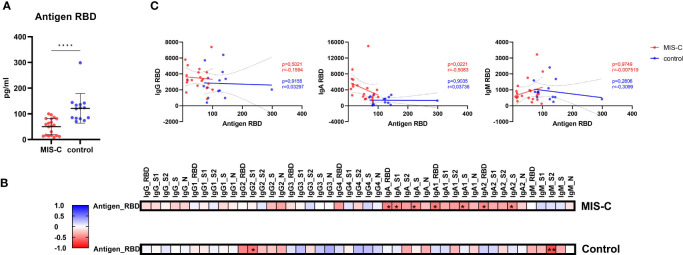
Antigen RBD dosage in serum measured by ELISA and correlations with antibody responses. Concentration of circulating RBD from SARS-CoV-2 **(A)** in MIS-C (red dots) and control children (blue dots) expressed in pg/ml. Means and standard deviations were represented. Correlation matrices **(B)** between the antigen RBD measured by ELISA and the measured isotypes and subclasses directed against SARS-CoV-2 receptor binding domain (RBD), S1 and S2 domain of the spike protein, trimerized full length spike (triS) and nucleocapsid protein (N), results were expressed with a color gradient for r coefficient and with stars for p values. Correlation dot plots **(C)** between the isotype, from left to right, IgG, IgA and IgM and the RBD antigen concentration in serum were represented in in MIS-C (red dots) and control children (blue dots). The regression line and the 95% confidence intervals dotted lines were represented on each graph with the calculated r and p values. **** 0.0001≤p.

Recently, a study reported higher spike antigen concentrations in the blood of children with MIS-C compared to controls due to increased permeability of the gut barrier leading to hyperinflammation (29). Therefore, we investigated whether we see a similar effect in our cohort. Interestingly, we detected significantly lower free RBD antigen concentrations in serum of MIS-C children compared to controls (**Figure 5A**). Next, we performed correlation analysis between antibody concentration and antigen, to investigate whether increased immune complex formation is the reason for lower free RBD antigen in MIS-C children. We found that IgA, but not IgG, anti-RBD antibodies showed a significant negative correlation in MIS-C children but not in controls (**Figures 5B, C**). These finding indicate that MIS-C children have a distinct IgA response that potentially leads to immune complex formation resulting in lower free RBD antigen.

## Discussion

To our knowledge, our study is the first to report a comprehensive analysis of the quality of the antibody response between MIS-C and age-matched control children at the same time after SARS-CoV-2 exposure. The key finding is that children with MIS-C developed a stronger IgA antibody response in comparison to children with uncomplicated COVID-19, while IgG and IgM responses are largely similar in both groups. We found a typical class-switched antibody profile with high level of IgG and IgA titers and a measurable low IgM titers due to relatively recent SARS-CoV-2 infection (one month). It is unclear if this difference in the IgA antibody response is associated with the onset or simply a consequence of the disease. It is tempting to speculate that this higher IgA antibody response is a consequence of the gastrointestinal inflammation, which is a prominent feature of MIS-C children (12). One third of the MIS-C children presented 7 days of fever and gastro-intestinal symptoms mimicking a viral gastroenteritis before consulting the hospital (30). This is associated with diffuse bowel wall thickening, with an intense terminal ileitis, mesenteric lymphadenitis, and ascites observed with abdominal sonography and computed tomography (30–32). Although SARS-CoV-2 was rarely detected in the stools and ileocolic biopsies from MIS-C patients by conventional methods, the presence of SARS-CoV-2 in the stools of MIS-C children has been measured by RT-PCR several weeks after initial SARS-CoV-2 infection or exposure. This suggests that the gut may be a chronic foci of viral infection in MIS-C (29, 33) and this is supported by detection of SARS-CoV-2 in the stools of children for a prolonged period of time after SARS-CoV-2 infection (34, 35). SARS-CoV-2 antigen is also detected 4 months post infection in intestinal biopsies of adults (36). A role for gut infection in MIS-C is further supported by a marked transmural lymphocytic inflammation and focal acute enteritis as well as numerous venous microthrombi in all layers of the ileum and signs of arteritis observed by microscopic examination of ileocolic specimen of MIS-C (32). A prolonged presence of SARS-CoV-2 in the intestinal tract induce the release of zonulin, an important regulator of the intestinal permeability through modulation of intercellular tight junctions, into the bloodstream (29). Other soluble marker of barrier dysfunction, such as the measure of syndecan 1 (SD1) in MIS-C children has already been assessed in a sub-group of our MIS-C children (37). These results have shown that MIS-C children present signs of endothelial glycocalyx lesions with an association between the degree of damage of glycocalyx and the severity of MIS-C. This would result in the release of a large amount of potential antigens and IgA from the gut lumen into the circulation. In line with this hypothesis, MIS-C children had increased LPS-binding protein and CD14 plasma levels, which are both markers of microbial translocation, compared to children with acute uncomplicated COVID-19 and pre-pandemic control children (29). Our observation that IgA to commensal bacteria is higher in MIS-C provide additional support for a role of sustained gastrointestinal infection by SARS-CoV-2 at the onset of the disease.

We observed higher IgA levels specific to SARS-CoV-2 spike in MIS-C, but a lower amount of free RBD antigen in serum of MIS-C children. In contrast to our results, another study recently found higher levels of SARS-CoV-2 S1 antigen, in children with MIS-C, compared to the children with acute uncomplicated COVID-19 and pre-pandemic controls but no difference in the IgA response (29). However, they assessed the IgA response and RBD antigen during acute infection in the control group but one-month post infection for the MIS-C group, while our study was designed to control for difference in the timing of sample collection post infection. They speculate that the circulation of high amounts of SARS-CoV-2 antigens in the bloodstream is thought to induce a hyperinflammatory response, due to the cytokine storm, in part related to the superantigen-like property of the spike protein (29). However, in the light of our findings using a time-matched control group that shows even higher free RBD antigen in serum of MIS-C children this interpretation seems less plausible. Instead, the negative correlation between RBD-specific IgA and free RBD antigen in MIS-C but not control children indicates that MIS-C children develop a distinct IgA antibody response after COVID-19. Further investigations on the IgA epitope pattern are therefore necessary. Whether this differential IgA response is causal or contributing to the development of MIS-C remains to be investigated.

We observed a more functional IgG antibody response in our MIS-C children, which is characterized by higher neutralizing capacity and avidity of IgG. IgG of MIS-C also had a stronger capacity to activate the complement system, whereas the capacity to activate neutrophils (ADNP) was similar to controls. Several hypotheses could explain these observations: First, children with MIS-C could have a prolonged exposure of immune cells to SARS-CoV-2 antigens, due to a sustained intestinal infection. Second, children with MIS-C may have a pool of pre-existing cross-reactive memory B cells originating from previous exposure to common cold coronaviruses. However, in our study, we did not observe higher antibody levels against cross-reactive SARS-CoV-2 derived antigens such as the S2 domain and N and did not find any difference in the antibody response against common cold coronavirus. Third, a difference in the microbiota of children with MIS-C, compared to children with uncomplicated COVID-19, could influence the intestinal inflammatory response and thereby alter the quality of the antibody response to SARS-CoV-2 generated locally. It is also possible that a continuous and prolonged exposure to the viral super-antigen-like and neurotoxin-like motifs in SARS-CoV-2 spike may promote autoimmunity leading to the development of MIS-C (38). It has been postulated that the super-antigen-like motif of the spike protein has a high affinity for binding T-cell receptors (TCRs) and MHC Class II proteins, inducing an exaggerated T-cell response. Indeed, it has been observed that MIS-C children developed a profound expansion of TCR β variable gene 11-2 (TRB11-2), which was associated with the severity of the MIS-C and with the concentration of the serum cytokines. Similarly, it has also been shown that SARS-CoV-2 spike were predominantly present in patients with post-acute sequelae of coronavirus disease 2019 (PASC) up to 12 months after diagnosis (39).

Our study has several limitations. The sample size of children with MIS-C is limited. MIS-C is a rare disease, and we only include children infected with antigenically similar SARS-CoV-2 variants in order to have the most comparable control group. We were also not able to perform a functional analysis of isolated IgA antibody, due to the low quantity of IgA in serum. We recognise that this is a limitation from our study. Indeed, it has been shown that IgA also had neutralising capacity (40), however, the strong correlation between the avidity of IgG antibodies and the neutralization capacity indicates that IgG and not IgA is the main contributor to neutralization.

In conclusion, even if it is still unclear why some children develop a MIS-C, we have shown that children with MIS-C produce higher titers of IgA antibodies, and IgG antibodies with higher functionality such as increased neutralizing activity, avidity, and inflammatory complement-binding capacity. We hypothesize that our findings reflect the local gastro-intestinal mucosal inflammation potentially induced by a sustained SARS-CoV-2 gut infection leading to continuous release of SARS-CoV-2 antigens.

## Data availability statement

The raw data supporting the conclusions of this article will be made available by the authors, without undue reservation.

## Ethics statement

The studies involving human participants were reviewed and approved by local Ethics committee of Geneva (CCER 2020 00835 and CCER 2020-00516). Written informed consent to participate in this study was provided by the participants’ legal guardian/next of kin.

## Author contributions

Conception of study: GB-R, AD, AM, BM, AT, CE, and C-AS. Patient recruitment: GB-R, NL, AL, SG, and FM; Experiments and analysis and interpretation of data: AT, BM, WA, PF, DG, and AP. Manuscript writing: AT, BM, GB-R, and AD; Review of manuscript: all authors. All authors contributed to the article and approved the submitted version.

## Supplementary material

Supplementary Figure 1 IgG antibodies directed against SARS-CoV-2 measured by ELISA. IgG antibody levels in MIS-C (red dots) and control children (blue dots) directed against SARS-CoV-2 derived S1 domain of the spike protein **(A)** and nucleocapsid protein (N) **(B).** Antibody titers are expressed as arbitrary units/ml (AU/ml) and were log10 transformed. The mean and standard deviations are represented.

Supplementary Figure 2 Linear regression analysis between log10 transformed PRNT90 antibody titers and antibody avidity measured as the dissociation constant (k_off_). All samples from MIS-C and control children are included. The regression line and the 95% confidence intervals dotted lines were represented.
