# Supplementary figures and images for: Antibody response in children with multisystem inflammatory syndrome related to COVID-19 (MIS-C) compared to children with uncomplicated COVID-19
Source: Front Immunol. 2023 Mar 15;14:1107156. doi: 10.3389/fimmu.2023.1107156 (PMC10050384; doi:10.3389/fimmu.2023.1107156)

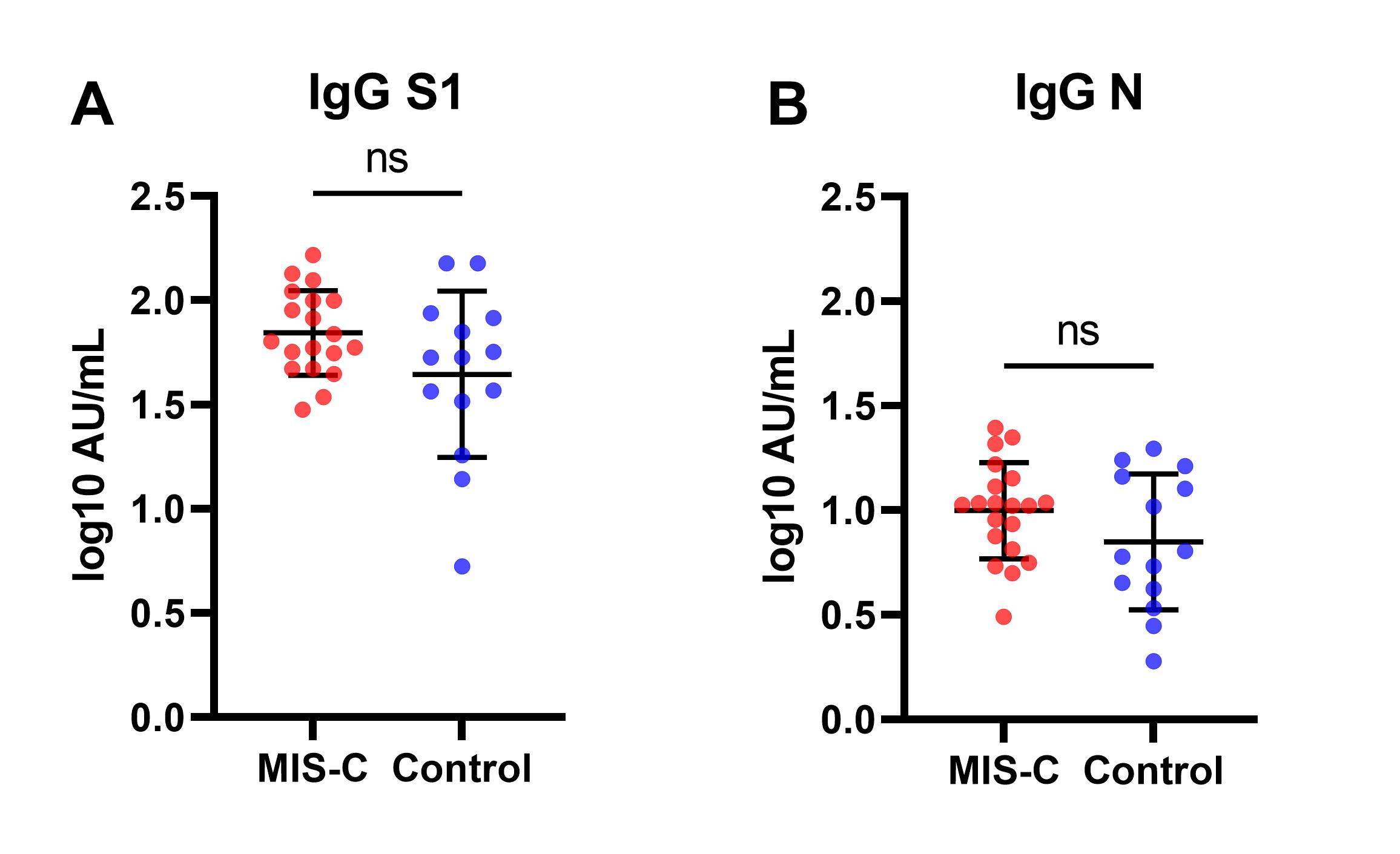

Supplement: Supplementary Figure 1 — IgG antibodies directed against SARS-CoV-2 measured by ELISA. IgG antibody levels in MIS-C (red dots) and control children (blue dots) directed against SARS-CoV-2 derived S1 domain of the spike protein (A) and nucleocapsid protein (N) (B). Antibody titers are expressed as arbitrary units/ml (AU/ml) and were log10 transformed. The mean and standard deviations are represented. [file Image_1.jpg]

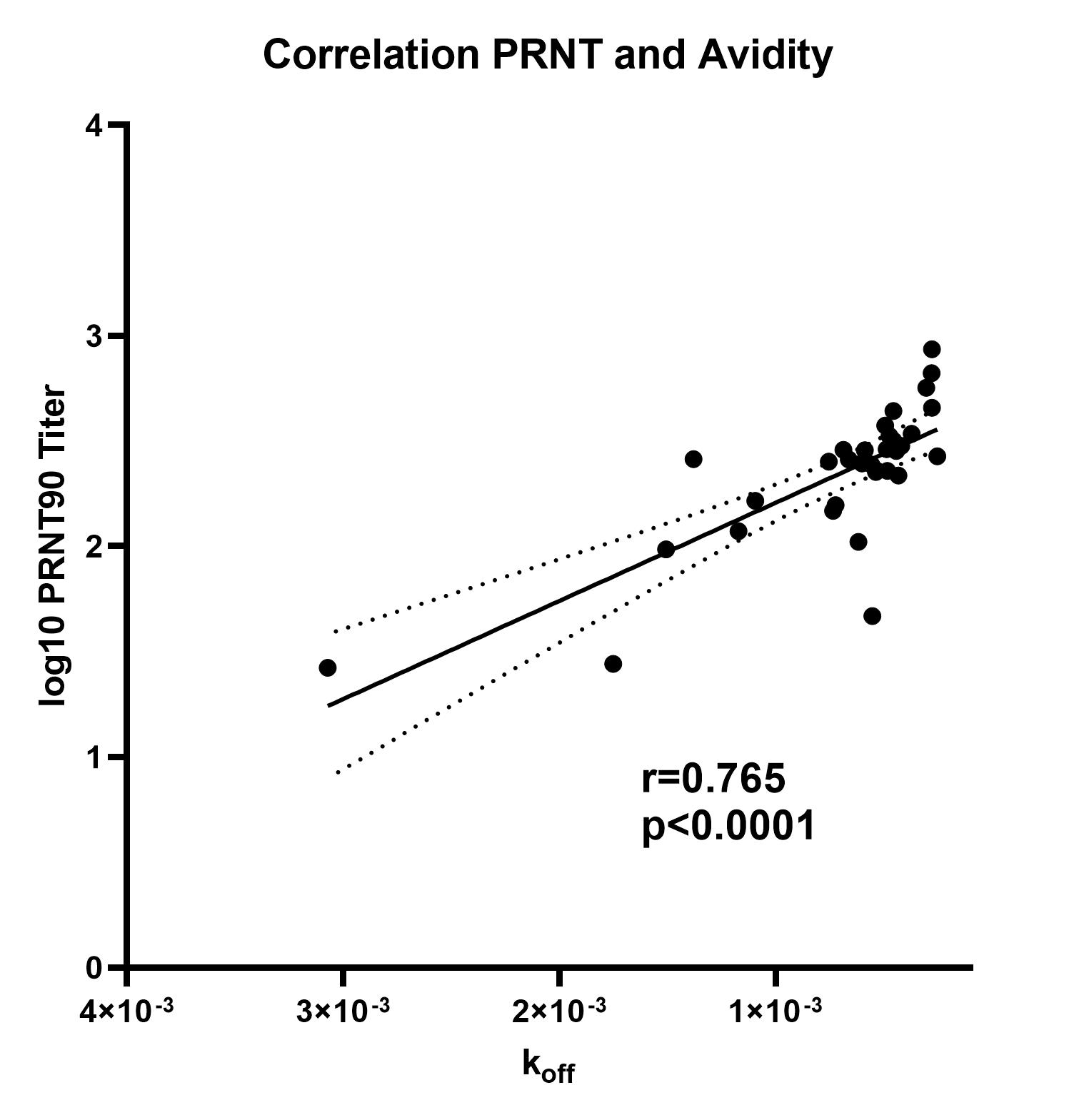

Supplement: Supplementary Figure 2 — Linear regression analysis between log10 transformed PRNT90 antibody titers and antibody avidity measured as the dissociation constant (koff). All samples from MIS-C and control children are included. The regression line and the 95% confidence intervals dotted lines were represented. [file Image_2.jpg]
